# Precision Anaesthesia: Advancing Patient-Centered Precision Care Through Repetitive Assessment of PROMs with the Safe Brain Initiative Approach

**DOI:** 10.4274/TJAR.2023.231420

**Published:** 2023-10-24

**Authors:** Başak Ceyda Meço, Ana Borda de Agua Reis, Joana Berger-Estilita, Karina Jakobsen, Neslihan Alkış, Finn Michael Radtke

**Affiliations:** 1 Ankara University Faculty of Medicine, İbn-i Sina Hospital, Department of Anaesthesiology and Intensive Care Unit, Ankara, Turkey; 2 Ankara University Brain Research Center (AÜBAUM), Ankara, Turkey; 3 University of Lausanne, Department of Anaesthesia, Lausanne, Switzerland; 4 University of Bern, Institute for Medical Education, Department of Anaesthesia; Hirslanden Salem-Spital, Institute of Anaesthesiology and Intensive Care, Bern, Switzerland; 5 Nykøbing Falster Hospital, Clinic of Anaesthesia, Nykøbing Falster, Denmark

**Keywords:** Neurocognitive disorders, patient-centered perioperative care, patient-reported outcome measures, postoperative delirium, precision anaesthesia, safe brain initiative

## Abstract

This article aims to introduce the Safe Brain Initiative (SBI) approach, focusing on collecting and leveraging Patient-Reported Outcome Measures (PROMs) to enhance patient-centred precision anaesthesia and prevent postoperative delirium (POD) and neurocognitive disorders (NCD). The SBI was implemented to systematically address the feedback gap in perioperative care by collecting and analysing real-world data. The initiative focuses on monitoring and preventing POD and NCD, providing effective anaesthesia care, assessing patient and team satisfaction, and evaluating environmental sustainability impact. Based on international guidelines, 18 core recommendations were established to address potential complications and challenges associated with anaesthesia. Preliminary results showed a notable reduction in POD and increased awareness among anaesthesia team members regarding PROMs. The SBI approach demonstrated significant benefits during emergency situations, such as the February 2023 earthquake in Turkey, by providing crucial support and comfort to victims requiring multiple surgical interventions. The SBI presents an innovative, cost-effective, and patient-centred approach to perioperative care. By integrating PROMs and systematic feedback mechanisms, the SBI aims to expedite the advancement of efficient, patient-centered precision perioperative care, improve patient outcomes, and elevate the quality of care. The initiative has shown promising results, and its adoption is growing globally. Collaboration among healthcare providers, researchers, and patients is crucial in shaping the future of anaesthesia practice and further improving patient outcomes. Turkish hospitals are encouraged to join the SBI to benefit from international collaborations and contribute to positive change in perioperative care standards. The SBI project significantly advances precision anaesthesia, emphasising personalised care and patient well-being.

**Main Points**

• The Safe Brain Initiative (SBI) is an evidence-based project that enhances patient-centred precision anaesthesia by integrating Patient-Reported Outcome Measures to monitor and prevent postoperative delirium and neurocognitive disorders.

• The SBI employs a comprehensive care bundle comprising 18 core recommendations derived from international guidelines, aiming to address potential complications associated with anaesthesia and surgery, such as postoperative pain, nausea, and vomiting.

• Preliminary results from the SBI project have shown a notable reduction in postoperative delirium rates and increased awareness among anaesthesia team members regarding patient-related outcomes, indicating the initiative’s effectiveness.

• The SBI project has gained global traction, with multiple hospitals from various countries participating in the initiative, fostering collaboration, knowledge exchange, and shared experiences to elevate the standards of perioperative care.

• By providing healthcare providers anonymous feedback regarding their patients’ outcome metrics, they can actively involve patients in their care, leading to better overall health outcomes, increased patient engagement, and reduced costs, contributing to improved patient experiences and continuous quality improvement.

## Introduction

Acquiring systematic feedback on Patient-Reported Outcome Measures (PROMs) remains a significant challenge for healthcare professionals and hospital administrators.^1^ PROMs encompass a wide range of patient-reported outcomes related to experiences, symptoms, and functional outcomes following medical interventions, including anaesthesia and surgery.^2,3^ Integrating PROMs as a vital component of precision care enables healthcare providers to gather comprehensive patient information directly, leading to personalised treatment decisions and interventions.^4^ This patient-centred approach enhances care quality by tailoring interventions, including the targeted application of medical technology, optimising treatment plans (e.g. fine tuning SOPs), and improving health outcomes based on a deeper understanding of individual needs and preferences.^5,6^

Ensuring the integrity of PROMs necessitates direct acquisition from patients without any interpretation by the clinical team or others. This approach is particularly critical in the postoperative setting, where an impartial assessment-uninfluenced by the experiences of individual service providers-is key to advancing precision care. It assists in detaching the influence of personal or departmental traditions and beliefs from the precision of PROMs and Postoperative Delirium (POD) assessments.

Ideally, these measurements should evaluate and classify patients’ health and functional status and the consequences associated with perioperative care. By doing so, they furnish a comprehensive assessment of care quality and side effects.^2,3,7^ Such a comprehensive and unfiltered view ensures accurate measurement and can contribute to continuous improvements in the quality and personalisation of patient care.

We introduced the Safe Brain Initiative (SBI) project^8^ to systematically address this feedback gap by collecting and leveraging real-world health outcome data to monitor, visualise, and enhance patient-centred care and preventive outcomes. The SBI initiative focuses on four key areas:

1. Acquiring PROMs to monitor and prevent POD/neurocognitive dysfunction (SBI-Core).

2. Facilitating strategic guidance for patient-centred anaesthesia management through the SBI-Muda initiative. This project seeks to optimise perioperative efficiency, mitigate POD/neurocognitive dysfunction, and elevate patient-reported outcomes. It provides weekly dashboard updates offering insights into OR and hospital metrics - encompassing e.g. start times or delays, suture-to-incision intervals, time expended in the post-anaesthesia care unit (PACU), and duration of postoperative hospital stays - all of which are contextualised alongside individual core outcomes.

3. Assessing patient and team satisfaction with perceived care quality (SBI-Us).

4. Evaluating and reducing environmental sustainability impact (SBI-Green).

**Figure 1:**
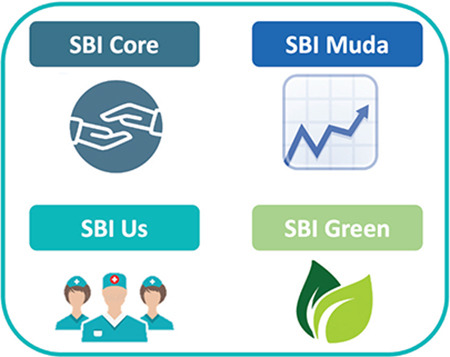
Four Focus areas of the Safe Brain Initiative.

By focusing on these areas, the SBI aims to strengthen the perioperative healthcare system, improve patient outcomes, and advance the understanding and practice of precision anaesthesia (Figure 1).

## PROMs in Perioperative Care

Within the sphere of anaesthesia, PROMs have an amplified importance compared to other specialties.^3,7^ This distinction arises from anaesthesia’s direct impact on patients’ immediate experiences and subsequent outcomes during and post-surgery.^9^ Critical PROMs to consider in a postoperative environment encompass postoperative pain, postoperative nausea and vomiting (PONV), thirst, stress and anxiety, overall well-being, and odynophagia (resulting from airway manipulation). In a broader context, outcomes like POD and perioperative neurocognitive disorders (PND) warrant close attention.

However, these outcomes are often monitored sporadically or superficially, neglecting the crucial need for continuous quality enhancement and precision in anaesthesia care.^10^ When the assimilation of individual and local patient outcome data is omitted, healthcare professionals forfeit a realistic appraisal of their individual or departmental methodologies’ efficacy. Moreover, the lack of structured, anonymous individual feedback deviates from the primary goal of anaesthesia: prioritising patient well-being. Developing comprehensive evaluation and feedback systems to address these issues is imperative, paving the way for effective learning trajectories and promoting sustained quality improvement.^11^

The consistent assessment of PROMs during the early postoperative period is paramount for several reasons.^7^ The fluctuating nature of recovery demands regular evaluations to document evolving symptoms, pain thresholds, functional status, and overall health. By closely monitoring PROMs, healthcare providers can swiftly identify escalating symptoms or complications, thus enabling timely interventions and optimising patient care.^12^ Furthermore, continual assessments enable the evaluation of treatment effectiveness over a given period, guaranteeing the achievement of desired outcomes and offering the chance for necessary modifications. By adapting care planning based on symptom changes and recovery progression, this personalised approach augments precision and individualisation in patient care.^13^ The assessment process also encourages patients to be more involved, fostering a sense of engagement and facilitating active participation in their recovery process.^14^ Regular evaluations provide patients with a platform to voice their worries, pose questions, and participate in shared decision-making, enhancing the overall patient experience in the long run.

### What is the SBI?

The SBI provides an evidence-based bundle of care to monitor and improve PROMs in Anaesthesiology and to prevent and reduce POD and PND in the perioperative period.^8^ It is a 360° concept for implementing and operationalising real-world evidence, with a data-driven dashboard solution for systematic feedback to healthcare personnel. With its comprehensive approach across the four-level domain, the SBI aims to leverage real-world healthcare outcomes data to monitor, visualise, and enhance preventive patient-centred routine care and outcomes.

**Figure 2:**
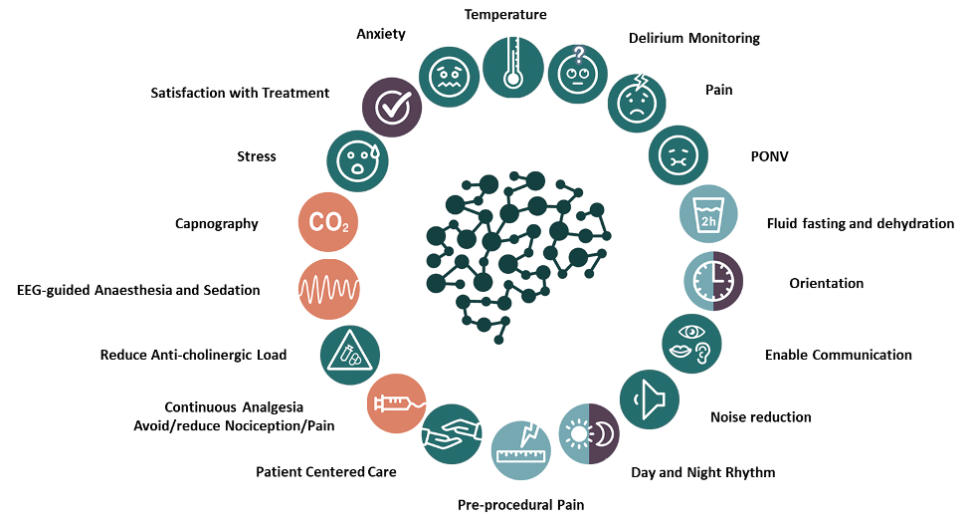
A multicomponent evidence-based approach: The 18 SBI core recommendations. SBI, Safe Brain Initiative, EEG: electroencephalography.

The SBI incorporates an extensive care bundle comprising 18 core recommendations (Figure 2). These recommendations primarily focus on non-invasive interventions to detect, prevent, and reduce adverse outcomes, including POD, PND, PONV, perioperative stress, perioperative anxiety, inadequate pain/nociception management, and patient discomfort. By implementing these recommendations, the SBI aims to address and mitigate potential complications and challenges associated with anaesthesia and surgery, ultimately improving patient outcomes and enhancing the overall surgical and perioperative experience.

1. Delirium monitoring: Implement measures to monitor and detect delirium in the perioperative period.

2. Preoperative pain: Address and treat pain before surgery to improve patient experience.

3. Stress: Employ strategies to lessen perioperative stress, fostering a more tranquil patient environment.

4. Anxiety: Recognize, address and reduce perioperative anxiety in patients through appropriate interventions and support.

5. Oral fluid fasting duration: Curtail unnecessary pre and post-surgery fasting periods.

6. PONV: Implement and fine-tune preventative measures and treatments for nausea and vomiting post-surgery.

7. Postoperative pain: Evaluate and effectively finetune preventive and postoperative pain treatment.

8. Facilitate communication: Ascertain that patients bring their dentures, hearing aids, and glasses to the Operating Room, PACU, and throughout the perioperative pathway, where applicable. This strategy aids in maintaining orientation and fostering and enhancing patient-centred communication between healthcare professionals and patients, thereby ensuring a transparent and effective exchange of information.

9. Patient-centered clinical practice: Emphasize a patient-centred approach where the patient’s preferences, needs, and values are considered and respected.

10. Anticholinergic influence: reduce the use and influence of anticholinergic medications on cognitive function where possible.

11. Electroencephalography (EEG) monitoring: Utilise the patient’s brain activity using EEG to individualise and finetune sedation application and to detect and prevent adverse neurological events.

12. Continuous analgesics and nociceptive monitoring: Use continuous analgesia techniques (e.g. remifentanil), and validated nociceptive monitoring to finetune and manage nociception/pain during and after surgery.

13. Use of Capnography in sedated patients: Implement capnography under sedation to ensure adequate ventilation and detect possible complications immediately.

14. Circadian rhythm: Support the patient’s circadian rhythm and incorporate strategies to support the natural sleep-wake cycle during the perioperative period.

15. Patient satisfaction: Measure and address patient satisfaction to improve the quality of care provided for all relevant perioperative care periods.

16. Noise: Minimize noise levels in the perioperative environment to promote a calmer and more comfortable patient and staff atmosphere and interaction.

17. Orientation: Minimize any potential impacts on patient orientation resulting from the side effects of perioperative care. Strive to maintain or enhance a patient’s baseline orientation concerning spatial awareness, temporal comprehension, and personal data throughout the perioperative period, except during anaesthesia.

18. Temperature: Maintain proper perioperative temperature management to prevent hypothermia or hyperthermia and promote patient comfort.

The interventions recommended by the SBI are designed to minimise or alleviate the iatrogenic burden on patients’ postoperative outcomes. These recommendations are derived from international guidelines or expert consensus and aim to enhance the patient’s perioperative experience, minimise complications, and promote positive outcomes. By implementing these non-invasive measures, healthcare providers can contribute to safer, more patient-centred perioperative care. The SBI recognises the importance of aligning interventions with evidence-based guidelines to ensure optimal outcomes and improve the overall quality of care for patients undergoing anaesthesia and surgery.

### The SBI has the following objectives:

• To offer an innovative, non-profit solution that enables anaesthesiologists, anaesthesia nurses, and anaesthesia departments to access and review their patients’ actual results and complications. The primary focus of the SBI is on the systematic monitoring and prevention of POD and PND, as well as PROMs, particularly in elderly and frail adults.

• To provide educational support for the routine assessment of the effects of implemented, modified, or newly introduced prevention and/or treatment strategies. These effects will be compared and analysed using high-quality, real-world data, allowing healthcare professionals to evaluate the impact of their interventions.

• To establish a solid foundation that enables healthcare professionals at the individual and departmental levels to embrace a continuous quality improvement process. The SBI aims to create an environment where healthcare providers can learn from real-world outcomes, identify areas for improvement, and implement changes that enhance the overall quality of care provided.

By achieving these objectives, the SBI strives to advance anaesthesia care, promote patient safety, and facilitate ongoing improvements in patient outcomes and experiences.

Patient-centred models of care, such as the SBI, are cost-effective and improve outcomes by actively involving patients in health decisions and ensuring their preferences are heard and acted upon.^6,13,15^ This approach leads to better overall health outcomes, increased patient engagement in their own care, and reduced costs.^16,17,18^

### The Impact of the SBI on Perioperative Care and Patient Support during Emergency Situations

Instituted in 2020 at Ankara University’s Faculty of Medicine, the SBI overcame initial implementation hurdles to evolve into a crucial instrument in enhancing the calibre of patient-centric perioperative care. To date, the SBI has enrolled over 5,000 surgical patients in Ankara University alone, showcasing a significant decline in the rates of postoperative delirium and augmenting the consciousness of anaesthesia team members about patient-related outcomes.

Beyond its routine benefits in perioperative practice, the SBI approach has demonstrated transformative impacts on improving the quality of perioperative care, particularly during the devastating earthquake that struck Turkey in February 2023. In the chaotic aftermath of the earthquake, the SBI strategy offered pivotal support and solace to victims necessitating multiple surgical procedures. In the context of perioperative care, from all SBI parameters, thirst emerged as a significant concern for these crush victims. The preoperative assessment of thirst was fundamental in addressing this issue effectively. By repetitively evaluating and quantifying the level of thirst preoperatively and postoperatively, the healthcare providers were equipped with valuable insights to tailor appropriate interventions and hydration strategies; and enhance patient comfort along with optimizing surgical outcomes and overall perioperative care.

Another significant concern arose from preoperative high levels of pain, which adversely affected patient comfort and surgical positioning. Efficient management of preoperative pain was crucial before the induction of anaesthesia, and the SBI approach played a central role in addressing this challenge.

Also, the systematic assessment of stress and anxiety levels and the well-being of patients emerged as a valuable non-pharmacological tool for premedication. Non-pharmacological management of high-stress levels was successfully attained through the implementation of interventions such as warm blankets, a hand-holding approach, and comforting talks. These strategies proved effective in mitigating psychological distress and enhancing patient well-being. This empathetic atmosphere nurtured by the SBI received high praise from patients, who affectionately referred to the anaesthesia team as “hand-holding doctors” due to their compassionate demeanor. This approach demonstrated instrumental in optimizing patient comfort and readiness for surgery, ultimately contributing to improved surgical outcomes and perioperative care. This strategy paved the way for fostering trust and mutual understanding between patients and the anaesthesia team, playing a critical role in safeguarding patients’ welfare and ensuring their safety during such crises.

### The Global Scope of the SBI Project

**Figure 3:**
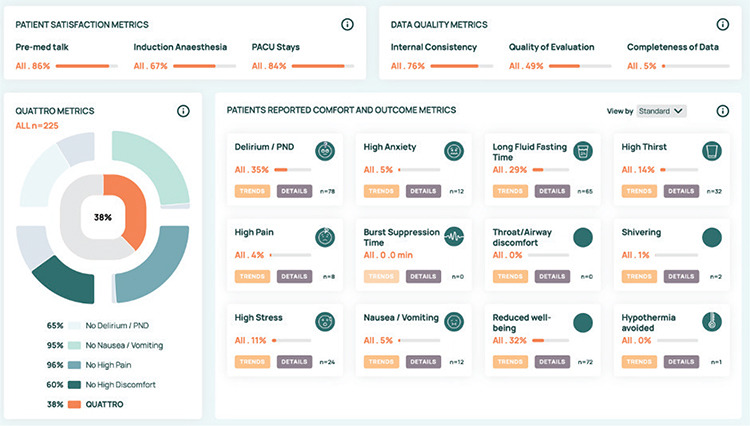
SBI dashboard example. SBI, Safe Brain Initiative.

The SBI project is an encompassing platform dedicated to facilitating scientific and practical benchmarking, promoting collaborative best practices, and inspiring a positive competitive environment for collective growth on various levels. It introduces a data-driven dashboard solution that furnishes systematic feedback for healthcare professionals (as depicted in Figure 3). Currently, the SBI network extends to multiple hospitals throughout Denmark, Germany, Switzerland, Saudi Arabia, and Turkey, fostering a diverse and cooperative landscape. The community of centres subscribing to the SBI is consistently expanding each month. Moreover, the European Society of Anaesthesiology and Intensive Care has recently endorsed the SBI by acknowledging it as a Research Group within the Society in recognition of its substantial value and prospective impact. Additional details and insights about the project can be retrieved from the SBI’s official website: safebraininitiative.com. This online platform is invaluable for those seeking comprehensive insights and updates regarding the initiative, its objectives, and ongoing developments.

Joining the SBI provides many benefits. These include access to resources and tools that help put best practices into action, ongoing monitoring of perioperative outcomes, and chances to participate in implementation projects with other hospitals. By combining PROM assessments from various hospitals during the early postoperative phase, we’re jointly working towards developing systematic strategies and components for precision anaesthesia. It’s important to recognize that this field is still nascent, and incorporating PROM assessments is a major step forward in enhancing our knowledge and application of precision anaesthesia.

We strongly encourage Turkish hospitals to take part in this initiative to reap the benefits of international cross boarder collaborations, the exchange of expertise, scientific contributions, and collective experiences with affiliate institutions. Membership in the SBI can bolster a hospital’s standing as a centre devoted to clinical excellence and patient-centred welfare.

The SBI is devoted to continuously enhancing and refining our methodologies and elements in providing bespoke and precise anaesthesia care. Collaborations among healthcare providers, researchers, and patients from diverse countries are pivotal in sculpting the future of anaesthesia practice and bolstering patient outcomes. We cordially invite all Turkish hospitals to participate in this meaningful international effort to foster beneficial changes and expansion in perioperative care.
